# Physical and Neurological Development of a Girl Born to a Mother with Methylmalonic Acidemia and Kidney Transplantation and Review of the Literature

**DOI:** 10.3390/children8111013

**Published:** 2021-11-05

**Authors:** Alessia Marcellino, Cristiana Alessia Guido, Silvia Bloise, Saverio Mallardo, Sara Isoldi, Emanuela Del Giudice, Anna Dilillo, Vanessa Martucci, Mariateresa Sanseviero, Donatella Iorfida, Alberto Spalice, Riccardo Lubrano

**Affiliations:** 1 Pediatrics and Neonatology Unit, Maternal-Child Department, Santa Maria Goretti Hospital, Sapienza University of Rome, 04100 Latina, Italy; marcellino.alessia@gmail.com (A.M.); silvia.bloise1989@gmail.com (S.B.); saverio.mallardo@gmail.com (S.M.); isoldi.sara@gmail.com (S.I.); emanuela.delgiudice@gmail.com (E.D.G.); Annadilillo83@gmail.com (A.D.); vany.mart@gmail.com (V.M.); mariateresa.sanseviero@yahoo.it (M.S.); donatella.iorfida@gmail.com (D.I.); 2 Pediatric Neurology Unit, Maternal-Child Department, Policlinico Umberto I, Sapienza University of Rome, 00185 Rome, Italy; cristiana.guido@uniroma1.it (C.A.G.); alberto.spalice@uniroma1.it (A.S.)

**Keywords:** methylmalonic acidemia, cognitive impairment, pregnancy, growth, neurological development, renal transplantation

## Abstract

Background: actual literature suggests that children of methylmalonic acidemia patients are mostly healthy, but data are only partial, especially regarding long-term outcome. Therefore, our aim was to evaluate the possible long-term neurological effects of fetal exposure to high levels of methylmalonic acid in a child of a renal transplant recipient. Methods: we retrospectively evaluated the clinical and neurological records of a girl whose mother is a kidney transplant recipient affected by methylmalonic acidemia. Subsequently, we compared our results with the ones already published. Results: the girl’s weight and stature were within the normal range in the first years of life but, starting from 4 years of age, she became progressively overweight. Regarding the neurodevelopment aspects, for the first time we performed a complete and seriated neuropsychological evaluation, highlighting a mild but significant weakness in the verbal domain, with a worsening trend at three-year revaluation. Conclusions: since children of MMA patients are exposed to methylmalonic acid, the efforts of the physicians caring for these children should be directed on careful evaluation of growth, prevention of obesity and regular neurological examination together with structured neuropsychological tests to achieve a better insight in possible complications of pregnancy in patients suffering from this condition.

## 1. Introduction

Methylmalonic acidemia (MMA) is an autosomal recessive disease due to an inborn error of intracellular cobalamin metabolism. It can be divided into four groups: cblA (MIM #251100), cblB (MIM #251110), cblD variant2 and mut (MIM #251000) [1]. The main symptoms are growth retardation, acute metabolic decompensation with acidosis, vomiting, dehydration, hepatomegaly, kidney injury until End Stage Renal Disease (ESRD) and psychomotor retardation with cognitive disfunction. Regarding the neurological involvement, the pathogenesis is still unclear; the central role of oxidative brain damage has been postulated due to the ability of methylmalonic acid to impair neuronal mitochondrial energy metabolism, glutamatergic transmission [2] and glutathione transport [3,4].

Nowadays, thanks to neonatal metabolic screening, early diagnosis and treatment are possible, with better outcomes. The fundamental treatment is a low-protein diet, according to proposed guidelines [5], with the goal of achieving at least the safe requirements established by the World Health Organization (2007). This usually leads to metabolic balance and nearly normal growth, despite elevated values of methylmalonic acid in urine and serum. As a consequence, a rising number of female patients achieve adulthood without severe disabilities and desire a regular life, including childbearing [6]. Nonetheless, it is known that some inborn errors of metabolic pathways may have a negative impact on both mother and child during pregnancy [7].

In the particular case of MMA, according to a recent review [8] of case reports, it seems that children of MMA patients are mostly healthy. Nonetheless, data regarding the possible long-term neurological effects of fetal exposure to high levels of methylmalonic acid are only partial. Given these premises, we retrospectively evaluated the clinical and neurological records of the girl previously described by our group [9] in order to add information about her long-term development.

## 2. Case Report

We report the case of a 10-year-old girl, born to a patient affected by CblA (homozygous pathogenic mutation c.586C>T (p.Arg196Term) in exon 4 of the MMAA gene) complicated with End Stage Renal Disease (ESRD) and renal transplantation at 16 years of age [10,11,12].

### 2.1. Pregnancy and Growth

The MMA patient became pregnant at the age of 29 years old. She and her husband had no family history of obesity or any developmental delay. Pregnancy was uneventful, except for mild hypertension in the third trimester, well controlled with β-blocker treatment. Before and during pregnancy the mother was on vitamin B12 and underwent a seriated evaluation of urinary methylmalonic acid levels in the second and the third trimester, showing levels much lower than in the literature (Table 1). At the 16th week of gestation, amniocentesis was performed and revealed elevated levels of methylmalonic acid in the amniotic fluid (127.3 µmol/L). The karyotype analysis showed a normal female fetus and the genetic test exhibited a heterozygous mutation c.586C>T (p.Arg196Term) in exon 4 of the MMAA gene, the same as described in the mother. On the other hand, the father did not carry any mutation in the same gene.

The girl was born at week 37 of gestational age by elective cesarean section; her weight was 2.48 kg (adequate for gestational age), length 53 cm, head circumference 34 cm and Apgar score was 8/9. Regular pediatric and neurological follow-up of the child until 30 months of age revealed no anomalies and no dysmorphism.

**Figure 1:**
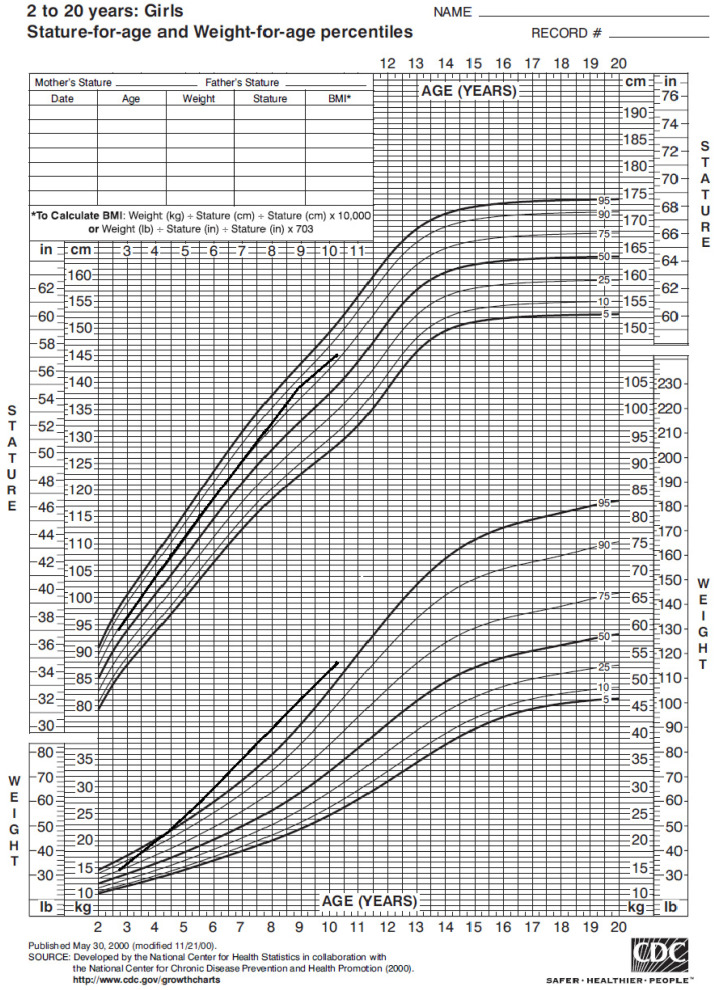
Statural–ponderal growth of our case from 2 to 10 years of age, according to CDC 2000 growth chart. Therefore, she started nutrition care visits; she showed mild hyperphagia, short satiation and low daily aerobic physical activity. In the past year she began a hypocaloric balanced diet and her BMI did not increase further. However, her parents did not give consent to obesity genetic tests.

**Figure 2:**
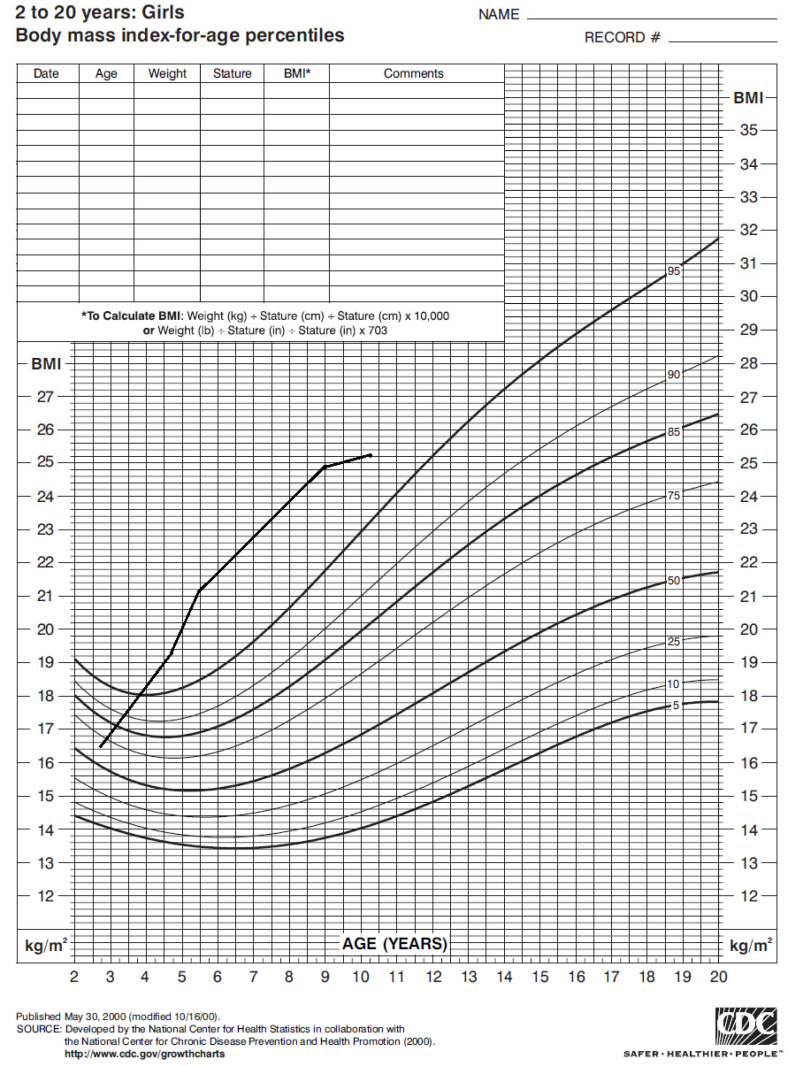
Body mass index of our case from 2 to 10 years of age according to CDC 2000 growth chart.

The girl’s weight and stature were within the normal range in the first years of life. Nonetheless, around 4 years of age her weight progressively increased (Figure 1), with a Body Mass Index (BMI) persistently above the 95° percentile (Figure 2).

### 2.2. Neurological Development

The girl’s family had a medium to high socio-cultural level, with great attention on education. The parents reported First Words (FW) at 7/8 months, Independent Walking (IW) at 13 months, sphincteric control at 2.5 years old and regular sleep. She attended a private kindergarten school without exhibiting any form of anxiety in separation from caregivers.

At the age of 7 years a learning disability screening revealed difficulties in reading and spelling, with need of a private speech therapy for a year. The deeper evaluation found difficulties mainly in the field of writing and logico-mathematical knowledge. The cognitive evaluation assessed through the Wechsler Intelligence Scale for Children—Fourth Edition (WISC-IV) test showed normal cognitive functioning with better performance in the verbal domain (FSIQ Tot 109; VCI 116; PRI 104; WMI 97; PSI 106). In particular, there was evidence of worsening in speediness performance (score −1.12: requires attention). The written exercise also showed a drop in the precision/correctness performance (score 4.74: prompt intervention), and regarding logical and mathematical skills, she presented difficulties in converting numbers (score 2: prompt intervention). In written exercises, written calculation and in mind math, she achieved scores that fell within the “In need of attention” range.

#### 2.2.1. Cognitive Profile

At the time of assessment, the girl was 10 years old and attended the fifth grade of primary school. The cognitive profile was evaluated by WISC-IV. She performed ranks in the Total IQ in the average range. Verbal, Verbal production, Perceptual-Reasoning, Abstraction and Working Memory abilities, immediate recall of auditory information and Processing Speed abilities were in the average range, together with the ability of working quickly with unusual materials; she also had average scores in divided and alternated attention and visual–spatial discrimination. On the other side, results showed scores below average in identification of meaningful relationships between concepts through categorization, ability below average in in-mind operations and return of auditory data after processing and scores much lower than average in social judgment and application of knowledge learned on the basis of consent and formal appropriateness. She resulted to have above average skills in the deductive ability to solve a new problem based on rules.

Thus, regarding the cognitive profile, the difference between the indices showed that the verbal domain represents a weakness point while the visual perceptual domain represents a key resource in the individual cognitive profile of the child.

#### 2.2.2. Behavioural Profile

The Child Behavior Checklist (CBCL) completed by the biological mother, showed the child’s Total Competence score in the clinical range below the 10th percentile for parents′ ratings of girls aged 6 to 11. The score on the Activities scale was in the clinical range below the third percentile, and the score on the Social scale was in the borderline clinical range (third to seventh percentiles), while the score on the School scale was in the normal range. On the CBCL problem scales, the child’s Total Problems and Externalizing scores were both in the normal range for girls aged 6 to 11; however, the Internalizing score was in the borderline clinical range (84th to 90th percentiles). Scores on all rated syndrome scales were in the normal range. On the DSM-oriented scales, the child’s scores on all rated scales were in the normal range.

Thus, as regard the behavioral profile, the girl’s biological mother reported problems of an internalizing nature.

#### 2.2.3. Profile of the Development and Adaptive Function

The development profile was measured by the Developmental Profile 3 (DP-3) questionnaire, compiled by the child’s mother. The overall development score was in the normal range (General Developmental score = 107, 95% confidence interval ± 10).

Adaptive behaviors were assessed through the Adaptive Behavior Assessment System—Second Edition (ABAS II); the questionnaire was completed by the mother of the child. It showed normal functioning in the conceptual main, but with scores under the standard in the Functional Academics, as the adaptive social domain; adaptive changes in particular showed a fall in the leisure. The Practical Adaptive Domain was in the “average” range.

All scores on development scales were average and, regarding adaptive behaviors, her scores indicated an average performance in adaptive abilities.

Given these results, the Neurology service proposed a brain MRI but the parents did not give consent.

## 3. Review

We performed a narrative review focusing on published children of MMA patients, in order to evaluate their somatic and neurological development and compare them to our case. Two different reviewers accessed Pubmed via the National Library of Medicine PubMed interface; the literature search was performed using the terms methylmalonic acidemia OR methylmalonic acid OR inborn errors of metabolism AND pregnancy OR children OR offspring. Further, they manually screened the reference lists of all included studies for additional data.

We identified 14 cases [6,8,12,13,14,15,16,17,18,19]. In Table 1 and Table 2 we reported all data regarding the aspects that could influence prenatal and postnatal growth and neurological development: pregnancy complications, treatments, protein intake, gestational age and weight at birth. In addition, we described the neurological outcome, the methods of neurological evaluation, mothers′ methylmalonic acid at first, second and third trimester and methylmalonic acid in the newborn’s urine and/or amniotic fluid.

## 4. Discussion

We decided to further investigate the long-term effect on growth and neurological development in a child born to a MMA patient. Our case resulted in line with previous literature [8] regarding statural and ponderal growth in the very first years of life (Table 1). Nonetheless, to the best of our knowledge, a pathological increase in BMI was never reported in offspring of patients with inborn errors of metabolism (IEM).

Regarding the neurodevelopmental aspects, a complete and seriated neuropsychological evaluation highlighted, in our patient, a mild but significant weakness in the verbal domain, with a worsening trend at three-year revaluation. As reported, literature lacks data in long-term follow-up even in the case of other IEMs [20,21] and in all the published cases of MMA children there are no reports of any neurological alteration.

These findings are unexpected, considering the high levels of methylmalonic acid in mothers′ blood and/or urine and/or amniotic fluid (Table 1) due to mothers′ disease. In addition, three authors [9,14,15], found increased levels of methylmalonic acid also in newborn’s serum or urine in the first hours of life (Table 1), attesting the actual exposure of the fetus.

It is known that methylmalonic acid has a negative effect on the neuronal energy metabolism, in particular respiratory chain in mitochondria, as demonstrated on murine models [4,22], leading to oxidative stress and subsequentially to necrotic and apoptotic cell death in cultured neuronal cells [23,24,25,26]. Moreover, Kolker et al. reported direct neuronal damage in vitro on chick embyo thelencephalos [27] due to methylmalonic acid. Finally, it has been demonstrated that the neurotoxicity is due also to direct damage on astrocytes and oligodendrocytes with activation of microglia in Knock-out (KO) murine models [28].

A possible explanation for the discordance between in vitro studies/animal models and case reports could be the delayed maturation of mitochondrial enzymes in the human fetus. Lubrano et al. [12] hypothesized that fetal neuronal cells are protected during pregnancy, until the very last weeks of gestation, from the toxic effect of methylmalonic acid thanks to the immaturity of mitochondrial enzymes, with milder and late neurological involvement in term babies. In fact, Wenchich et al. [29] and Honzik et al. [30] revealed that the efficiency of respiratory chain enzymes in human muscle of preterm neonates rises gradually between the 28th and 40th weeks. Additionally, Ravera et al. [31] showed that the energy metabolism in mesenchymal stem cells of a preterm umbilical cord is mainly anerobic due to inefficiency of mitochondrial enzymes, giving the tissues higher protection against oxidative stress without loss of the energy needed for growth and development. In addition, in a recent murine model [28] a significant lower accumulation of methylmalonic acid has been demonstrated in more immature KO brains.

In our case, since obesity developed rapidly from 4 years of life, we hypothesized that methylmalonic acid exposure during fetal life could lead, as previously mentioned, to alterations in mitochondrial dynamics and microglia activation in the hypothalamus. In fact, the microglial system is important to achieve metabolic control [32] and a recent review [33] highlighted the central role of mitochondrial imbalance in obesity and metabolic syndrome.

Nonetheless, our findings are difficult to interpret because of lack of data reports of similar cases. There are no published records of BMI monitoring of the 13 children described in literature and the neurological follow-up seems to be a short-term one, involving mostly preschool children (Table 1). In 1 case out of 13 there are no records about neurological development. Only two cases reported by Raval et al. [8] were evaluated at school age (10 and 14 years old, respectively) but it is not clear if these children underwent a full neurological visit together with neuropsychological tests. In addition, only one author [13] specified the neuropsychological tests used during follow-up.

These features and the well-known sensitivity of the WISC scales as neuropsychological tests could explain our unique findings. We think that since during the last months of pregnancy, the fetal brain undergoes significant growth, cortical folding and maturation of enzymatic activities [34,35], a late effect of methylmalonic acid on these events could be possible, when the protection of immature mitochondrial enzymes is over, with mild but persistent damage.

In conclusion, we suggest that since children of MMA patients are exposed to high levels of methylmalonic acid and it seems to have a negligible teratogenic effect, the efforts of the physicians caring for these children might be directed towards careful evaluation of growth, prevention of obesity and regular neurological examination together with structured neuropsychological tests, with long-term revaluations. Given the actual rarity of these case reports, to confirm or refute our hypothesis, it would be desirable to perform multicentric studies involving gynecologists, pediatricians and neonatologists, and also to achieve better insight in possible complications of pregnancy in patients suffering from MMA.

## Figures and Tables

**Table 1:** Characteristics of the published mothers with methylmalonic acidemia.

Published Case	Type of MMA	Pregnancy Metabolic Complications	Adjunctive Therapy	Protein Intake	Obstetrical/ Delivery Complications
*Diss 1995*	mut^0^	Hyperemesis	Carnitine	3.6 → 4.1 → 1.6 g/kg/die	None
*Wasserstein 1999*	mut^−^	None	Oral bicarbonate Amoxicillin/Metronidazole	40–55 g/die	pPROM Pre-eclampsia
*Deodato 2002*	mut^−^	None	Carnitine Vitamin B12 IM	1.5 g/Kg/die	None
*Boneh 2002*	CblA	None	Vitamin B12 IM	Reported as “normal”	Fetal distress
*Adeyemi 2004*	mut^−^	IV fluids once	Vitamin B12 OS Carnitine	Reported as “low”	pPROM Prepartum Chlamydia Infection Chorioamnionitis
*Langendonk 2011*	N/A	Hyper ammoniaemia twice	Carnitine Vitamin B12 IM Vitamin B9	45–60 g/die	None
*Langendonk 2011*	mut^−^	Poor treatment compliance	Carnitine Vitamin B12 IM	35 g/die	IUGR Difficulty feeding
*Lubrano 2013*	CblA	None	None	Reported as “low”	None
*Jacquemyn 2014*	CblA	None	Carnitine Vitamin B12 OS	1 g/Kg/die	None
*Raval 2015*	mut^−^	IV Fluids twice	Carnitine	64 g/die	Fetal distress Post-partum infection
*Raval 2015*	CblA	None	N/A	45 g/die	Pre-eclampsia Fetal distress
*Raval 2015*	CblA	None	Carnitine, Vitamin B12 IM	45 --> 80 g/die	Pre-eclampsia Gestational diabetes pPROM Fetal distress
*Raval 2015*	mut^−^	None	Carnitine	47 g/die	None
*Wilcox 2018*	N/A	None	N/A	N/A	None

**Legend:** Methylmalonic acidemia (MMA), maternal blood (MB), maternal urine (MU), amniotic fluid (AF), adequate for gestational age (AGA), small for gestational age (SGA), preterm premature rupture of membranes (pPROM), cord blood (CB), neonatal blood (NB) neonatal urine (NU).

**Table 2:** Characteristics of the published children of methylmalonic acidemia patients.

Published Case	Gestational Age	Birth Weight	Neurological Outcome of the Child	Methods of Evaluation	Methylmalonic Acid MB µmol/L MU mmol/mol Cr AF µmol/L	Newborn’s Levels of Methylmalonic Acid CB µmol/L NB µmol/L NU mmol/mol Cr
*Diss 1995*	Term	3500 g (AGA)	Normal at 3 years	Denver Developmental Screening Tests	1st trim. 16.4 (MB) 2nd trim. 8.2 (MB) 3rd trim. 9 (MB) 1st trim. 2.59 (MU) mmol/24 h 2nd trim. 1.29 (MU) mmol/24 h 3rd trim. 0.78 (MU) mmol/24 h N/A (AF)	N/A
*Wasserstein 1999*	36 weeks + 4 days	3220 g (AGA)	Normal at 1 year	N/A	484 (MB) 4.98 (MU) 608.7 (AF)	58 (CB) Undectable at 5 days (NB)
*Deodato 2002*	38 weeks	2940 g (AGA)	Normal at 2 years	N/A	N/A (MB) 1st trim. 17049 (MU) 2nd trim. 4177 (MU) 3rd trim. 3236 (MU) 305 (AF)	200 (CB) 1129 (NU) at birth 82 (NU) at 48 h
*Boneh 2002*	36 weeks	N/A	N/A	N/A	N/A (MB) 210-385-600 (MU) N/A (AF)	N/A
*Adeyemi 2004*	34 weeks	1900 g (AGA)	Normal at 5 months	N/A	N/A (MB) Reported as “raised” (MU) N/A (AF)	Undectable (NU)
*Langendonk 2011*	38 weeks	2850 g (AGA)	Normal at 5 years	N/A	N/A	N/A
*Langendonk 2011*	35 weeks	1530 g (SGA)	Normal at 1 year	N/A	N/A	N/A
*Lubrano 2013*	37 weeks	2480 g (AGA)	Normal at 3 months	WISC	N/A (MB) 1st trim. N/A (MU) 2nd trim. 72 (MU) 3rd trim. 52 (MU) 127.3 (AF)	7 (NU) at 48 h 0.2 (NB) at 28 days
*Jacquemyn 2014*	40 weeks	3300 g (AGA)	Reported as “normal”	N/A	N/A	N/A
*Raval 2015*	38 weeks	3288 g (AGA)	Normal at 10 years	N/A	N/A	N/A
*Raval 2015*	42 weeks	3714 g (AGA)	Normal at 14 years	N/A	N/A	N/A
*Raval 2015*	32 weeks	1459 g (AGA)	Normal at 3 years	N/A	1st trim. 39 (MB) 2nd trim. 45 (MB) 3rd trim. 15 (MB) N/A (MU) N/A (AF)	N/A
*Raval 2015*	39 weeks	3095 g (AGA)	Normal at 3 months	N/A	N/A (MB) 765 (MU) N/A (AF)	N/A
*Wilcox 2018*	N/A	N/A	N/A	N/A	N/A	N/A

**Legend:** Methylmalonic acidemia (MMA), maternal blood (MB), maternal urine (MU), amniotic fluid (AF), adequate for gestational age (AGA), small for gestational age (SGA), preterm premature rupture of membranes (pPROM), cord blood (CB), neonatal blood (NB), neonatal urine (NU).
